# Saphenous neuropathy in a patient with low back pain

**DOI:** 10.1186/1749-7221-5-2

**Published:** 2010-01-16

**Authors:** Tannaz Ahadi, Gholam Reza Raissi, Mansoureh Togha, Parisa Nejati

**Affiliations:** 1 Physical Medicine and Rehabilitation Department, Iran University of Medical Sciences, Firoozgar hospital, Tehran, Iran; 2 Neurology Department, Tehran University of Medical Sciences, Sina Hospital, Tehran, Iran; 3 Sports Medicine, Iran University of Medical Sciences, Iran

## Abstract

Saphenous nerve, a pure sensory nerve, may compromise as a result or complication of a surgical procedure or secondary to trauma or insidiously. We present a male patient with low back pain concomitant with pain in medial portion of left thigh in addition to pain and numbness in medial part of leg and inferior part of patella after a strenuous activity. Preliminary diagnosis suggested that the patient had radiculopathy but electrodiagnostic tests revealed the absence of left saphenous response both in medial leg and infrapatellar region, while normal findings were recorded from right side. Needle electromyography in L4 innervated muscles were normal. The patient had saphenous nerve entrapment in left thigh. Two months later symptoms relieved with conservative therapy.

## Background

Saphenous nerve is a pure sensory nerve that is made up of fibers from L3 and L4 spinal segments [1]. Because of its long course, it can become entrapped in multiple locations but mostly in two sites: the first site is in adductor canal after the saphenous nerve has split from the femoral artery and courses independently through the fascial channel in the adductor canal, the second site is at the exit point of the saphenous nerve distally in the thigh, where it penetrates the fascial tissue between the sartorius and gracilis muscles [2]. This problem may arise as a result or complication of a surgical procedure or secondary to trauma or it may arise insidiously. Primary saphenous neuropathy is uncommon [3]. The differential diagnoses of saphenous entrapment are: patellofemoral disorders, suprapatellar plica, tear of medial meniscus, pes tendonopathy, osteochondritis dissecans, nonspecific synovitis and reflex sympathetic dystrophy [4]. We present a patient with low back pain that received recommendation for surgery of radiculopathy but had saphenous nerve entrapment in left thigh.

## Case presentation

The patient is a 32-year old athlete man who complained of low back pain concomitant with pain in medial portion of left thigh in addition to pain and numbness in medial part of leg and inferior part of patella. After a strenuous activity, he felt pain in low back area and severe local pain in midportion of thigh accompanied by numbness of infrapatellar area and medial part of leg. His low back pain was reduced after consumption of NSAIDs but numbness continued. In physical examination, sensation to light touch and pinprick in infrapatellar and medial part of left leg was impaired. Manual muscle test and muscle stretch reflexes were normal, and the patient had no pain in straight as well as reversed straight leg raise. MRI of lumbosacral region showed bulging of the L4, L5 and S1 discs.

With impression of radiculopathy, surgical intervention for discopathy was recommended for the patient. Electrodiagnostic tests performed in standard protocol [5] by first and second author revealed absence of left saphenous response both in medial leg and infrapattellar region while normal findings were recorded from right side. Needle electromyography was normal in all tested muscles including quadriceps and paraspinal muscles. Neural block was recommended to the patient but he did not accept. Conservative management including Gabapentin was prescribed. Patient's symptoms relieved after 2 months, and six months later he had no symptom. He refused another Electrodiagnostic study.

## Conclusion

Clinical symptoms and electrodiagnostic findings revealed saphenous nerve entrapment at adductor canal or above this region. Saphenous neuropathy usually presents with pain[4] however, sensory complaints may be the presenting symptom. If L4 radiculopathies is caused by far lateral disc protrusion, sensory evoked response of saphenous nerve may be absent due to pressure on sensory ganglion [6]. In such cases, needle electromyography in L4 innervated muscles can differentiate between radiculopathy and saphenous neuropathy.

A thorough physical examination is mandatory in patients with low back pain and uncommon neuropathies like saphenous nerve entrapment must be considered.

## Consent

Written informed consent was obtained from the patient for publication of this case report.

## Competing interests

The authors declare that they have no competing interests.

## Authors' contributions

TA and GR contributed in electrodiagnosis testing. All authors contributed in taking patient history. Physical exam and preparation of the paper. All authors read and approved the final manuscript.
